# Validation of Echocardiographic Measurements in Patients with Pulmonary Embolism in the RIETE Registry

**DOI:** 10.1055/s-0043-1777765

**Published:** 2024-01-08

**Authors:** Mads Dam Lyhne, Behnood Bikdeli, David M. Dudzinski, Alfonso Muriel-García, Christopher Kabrhel, Teresa Sancho-Bueso, Esther Pérez-David, José Luis Lobo, Ángel Alonso-Gómez, David Jiménez, Manuel Monreal, the RIETE Investigators

**Affiliations:** 1 Department of Anesthesiology and Intensive Care, Aarhus University Hospital and Department of Clinical Medicine, Aarhus University, Aarhus, Denmark; 2 Cardiovascular Medicine Division, Brigham and Women's Hospital, Harvard Medical School, Boston, Massachusetts, United States; 3 Thrombosis Research Group, Brigham and Women's Hospital, Harvard Medical School, Boston, Massachusetts, United States; 4 Yale-New Haven Hospital (YNHH)/Yale Center for Outcomes Research and Evaluation (CORE), New Haven, Connecticut, United States; 5 Department of Cardiology, Massachusetts General Hospital, Boston, Massachusetts, United States; 6 Unidad de Bioestadística Clínica, Hospital Universitario Ramón y Cajal, Madrid and CIBERESP, Universidad de Alcalá, Madrid, Spain; 7 Department of Emergency Medicine, Centre of Vascular Emergencies, Massachusetts General Hospital, Boston, Massachusetts, United States; 8 Department of Internal Medicine, Hospital Universitario La Paz, Madrid, Spain; 9 Department of Cardiology, Hospital Universitario La Paz, Madrid, Spain; 10 Department of Pneumonology, Hospital Universitario Araba, Álava, Spain; 11 Department of Cardiology, Hospital Universitario Araba, Álava, Spain; 12 Respiratory Department, Hospital Ramón y Cajal and Medicine Department, Universidad de Alcalá, Madrid, Spain; 13 CIBER Enfermedades Respiratorias (CIBERES), Madrid, Spain; 14 Chair for the Study of Thromboembolic Disease, Faculty of Health Sciences, UCAM - Universidad Católica San Antonio de Murcia, Murcia, Spain

**Keywords:** echocardiography, right ventricular function, validity, pulmonary circulation, reliability

## Abstract

**Background** In acute pulmonary embolism (PE), echocardiographic identification of right ventricular (RV) dysfunction will inform prognostication and clinical decision-making. Registro Informatizado Enfermedad TromboEmbolica (RIETE) is the world's largest registry of patients with objectively confirmed PE. The reliability of site-reported RV echocardiographic measurements is unknown. We aimed to validate site-reported key RV echocardiographic measurements in the RIETE registry.

**Methods** Fifty-one randomly chosen patients in RIETE who had transthoracic echocardiogram (TTE) performed for acute PE were included. TTEs were de-identified and analyzed by a core laboratory of two independent observers blinded to site-reported data. To investigate reliability, intraclass correlation coefficients (ICCs) and Bland–Altman plots between the two observers, and between an average of the two observers and the RIETE site-reported data were obtained.

**Results** Core laboratory interobserver variations were very limited with correlation coefficients >0.8 for all TTE parameters. Agreement was substantial between core laboratory observers and site-reported data for key parameters including tricuspid annular plane systolic excursion (ICC 0.728; 95% confidence interval [CI], 0.594–0.862) and pulmonary arterial systolic pressure (ICC 0.726; 95% CI, 0.601–0.852). Agreement on right-to-left ventricular diameter ratio (ICC 0.739; 95% CI, 0.443–1.000) was validated, although missing data limited the precision of the estimates. Bland–Altman plots showed differences close to zero.

**Conclusion** We showed substantial reliability of key RV site-reported measurements in the RIETE registry. Ascertaining the validity of such data adds confidence and reliability for subsequent investigations.

## Introduction

Acute pulmonary embolism (PE) is a disease with risk of rapid deterioration and is potentially fatal. 1 2 A leading cause of death is acute RV failure due to an abrupt increase in pulmonary pressure which may lead to right ventricular (RV) dilatation and compromised RV systolic function. 2 3 4 Accordingly, transthoracic echocardiographic (TTE) identification of RV dysfunction is central for risk stratification to guide acute PE management. 1 5

Registries provide crucial information about epidemiology for acute PE. The Registro Informatizado de la Enfermedad TromboEmbolica (RIETE) registry 6 is the world's largest registry on patients with acute venous thromboembolism (VTE) and provides invaluable data on acute PE. However, echocardiographic parameters in registries, including RIETE, are site-reported. Therefore, there is inherent uncertainty about the reliability of these measurements across different sites that might undermine the results of analyses from the registries. The reliability of TTE data among PE patients in general is further challenged by the subjective nature of diagnosing RV dysfunction on TTE.

Considering the importance of the assessment of RV function in acute PE, we aimed to validate site-reported key RV echocardiographic measurements in the RIETE registry to ensure data reliability.

## Materials and Methods

### Data Source

RIETE is an ongoing worldwide multicenter registry including patients with objectively confirmed VTE. The methodology of the registry has been published previously. 6 The registry contains information on demographics, past medical history, laboratory tests, diagnostic workup, treatment, and follow-up of VTE patients. As of March 2023, 114,201 VTE patients have been covered, including 57,023 with PE.

### Ethical Considerations

Enrollment in RIETE is approved by local ethics committees at each participating site. The protocol was first approved by the Ethics Committee of Hospital Germans Trias i Pujol (number PR[AG]213/2020), and then in all participating centers. All enrollees in the registry provided informed consent according to requirements of local institutional review boards of participating centers. For the current study, recorded TTEs were transferred and stored securely in an anonymous manner according to data management agreement (Central Region Denmark, no. 1-52-81-5-22). The study applies to the Guidelines for Reporting Reliability and Agreement Studies (GRRAS) recommendations intended for the conduction and reporting of reliability studies. 7

### Echocardiographic Analyses

To investigate reliability of TTE data entered in the registry, we requested patient information and TTEs of randomly selected patients from three high volume-contributing sites, though included patients originated from two of the sites encompassing 4,085/47,342 (8.6%) of patients enrolled in RIETE. Sites were chosen based on their ability to secure independent ethics agreements for transfer of echocardiograms. Missing data from site-reported echocardiographic reports were nonimputed. RIETE does not have any requirements on who to perform the echocardiograms, nor does RIETE have an echocardiogram protocol though individual sites may have. The included echocardiograms were obtained between August 22, 2019 and September 13, 2021, and there were no changes in protocols during this time period.

Core laboratory analysis was performed using OsiriX MD (v. 13.0.2). All analyses were performed according to guidelines using an average of three cardiac cycles when possible. 8 9 Two observers independently analyzed the echocardiograms blinded to RIETE values and to clinical information. Measurements were performed whenever possible irrespective of any suboptimal angle as recordings were already acquired. Angle appropriateness was not assessed.

**Fig. 1:**
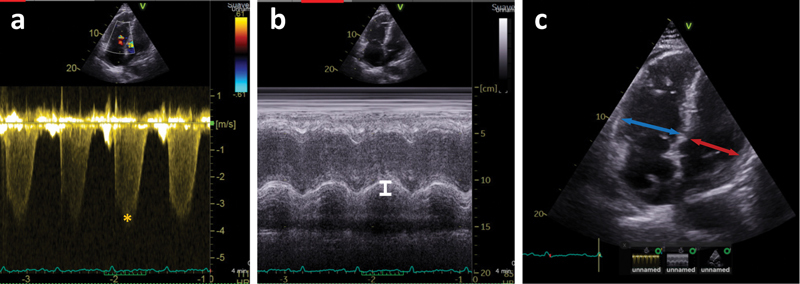
Illustrative examples of echocardiographic images. Representative echocardiographic images from an RIETE patient. Maximal tricuspid regurgitation gradient (TRG) is measured (asterisk in panel [ **a** ]) to calculate right ventricular (RV) systolic pressure. Tricuspid annular plane systolic excursion (TAPSE) is measured in M-mode recording ( **b** ) showing the longitudinal change of the RV. In panel ( **c** ), diameters of the RV in blue and the left ventricle in red are measured at the tip of the leaflets in end-diastole. RIETE, Registro Informatizado Enfermedad TromboEmbolica.

The following echocardiographic variables were assessed ( Fig. 1 ): tricuspid annular plane systolic excursion (TAPSE), tricuspid regurgitation gradient (TRG), inferior vena cava (IVC) diameter and collapsibility (below vs. above 50%), and end-diastolic RV diameter and left ventricular (LV) diameter in apical four-chamber view. We calculated the tricuspid regurgitation pressure gradient (TRPG) using Bernoulli's simplified equation. Right atrial pressure (RAP) was estimated according to practice guidelines by assessment of the inferior vena cava. 8 9 Pulmonary arterial systolic pressure (PASP) was calculated as TRPG + RAP, which is equal to right ventricular systolic pressure with the assumption of no pulmonary stenosis. We calculated TAPSE/PASP ratio and RV/LV ratio. According to TTE and PE guidelines, we used the following cutoffs for abnormality: TAPSE < 17 mm, RV basal diameter > 41 mm, RV/LV ≥ 1, PASP > 36 mm Hg, and tricuspid regurgitation velocity (TRV) > 2.9 m/s. 1 8 9

### Statistical Considerations

For an expected reliability of 80% (and a precision of 10%), we calculated that we would a need a total of 51 echocardiographic studies. 10 Intraclass correlation coefficients (ICCs) were interpreted as poor < 0; slight, 0.00 to 0.20; fair, 0.21 to 0.40; moderate, 0.41 to 0.60; substantial, 0.61 to 0.80; and almost perfect, 0.81 to 1.00. 11 12 We decided a priori to consider measurements to be reliable with correlation coefficients >0.6 (i.e., “substantial” or “almost perfect”).

We calculated correlation coefficients using Lin's method 13 between the two observers. To compare the core laboratory analysis with the RIETE registry, we averaged the two observers' measurements and then compared with the RIETE registry data calculating new ICCs. We also created Bland–Altman plots. To determine clinical relevance, we calculated the frequency of disagreement between the two observers and RIETE data on categorization of “normal” and “abnormal” of TTE findings and calculated kappa statistics for these categorical observations. For dichotomous variables, we did not calculate a two-observer mean value for the kappa index, but reported each of the observers against RIETE registry data. We investigated how often that change in categorization caused a change in clinical risk stratification according to guidelines. 1 Stata 17.1 (StataCorp LLC, TX) was used for all analyses.

## Results

We retrieved a total of 54 randomly chosen TTEs. Three did not have imaging clips of TAPSE, TRG or RV/LV diameter ratio, and were excluded. A total of 51 patients were included in the study. Patient characteristics are presented in Table 1 .

**Table 1:** Patient characteristics

Sex (male)	20 (39%)
Age	71 ± 15
Past medical history	
Angina or myocardial infarction	4 (8%)
Cerebral ischemia	4 (8%)
Arterial hypertension	29 (57%)
Heart failure	7 (14%)
Atrial fibrillation	1 (2%)
Chronic lung disease	7 (14%)
Cancer	9 (18%)
Risk factors	
Smoker	4 (8%)
Surgical intervention <2 months	4 (8%)
Immobility for more than 4 days <2 months	12 (24%)
History of DVT or PE	6 (12%)
Clinical presentation	
Heart rate, 1/minute	95 ± 21
Systolic blood pressure, mm Hg	135 ± 25
Pulse oximetry, %	91 ± 5
Risk stratification	
sPESI score ≥ 1	34 (67%)
ESC high-risk class	0 (0%)
ESC intermediate-high risk class	4 (8%)
ESC intermediate-low risk class	14 (27%)
ESC low risk class	9 (18%)
Insufficient information to provide ESC risk class	24 (47%)
PE localization	
Central PE, unilateral/bilateral	0 (0%)/5 (10%)
Lobar PE, unilateral/bilateral	8 (16%)/21 (41%)
Segmental PE, unilateral/bilateral	13 (25%)/31 (61%)
Subsegmental PE, unilateral/bilateral	88 (16%)/22 (43%)
Echocardiographic findings	
TAPSE, mm	21 ± 5
PASP, mm Hg	45 ± 13
TAPSE/PASP	0.51 ± 0.22
RV/LV ( *n* = 10)	1.0 ± 0.2

Abbreviations: DVT, deep venous thromboembolism; ESC, European Society of Cardiology; LV, left ventricular; PE, pulmonary embolism; PASP, pulmonary arterial systolic pressure; RV, right ventricular; sPESI, simplified Pulmonary Embolism Severity Index; TAPSE, tricuspid annular plane systolic excursion.

Data are presented as mean ± standard deviation or *n* (%) where appropriate.

TTE was performed within 1 day of PE diagnosis in 18 patients (35%), 2 days in 11 (22%), or ≥3 days in 17 (33%) patients. Information was missing in five (10%) cases. Two TTEs had missing information on TAPSE and TRG, whereas two other TTEs had missing information on RV/LV diameter ratios. Accordingly, we compared 49 TTEs between the two observers. Although RIETE data and core laboratory data had reasonable correlation for RV diameter, since such data were available in RIETE only for a few participants ( *n* = 5), we decided to remove it from formal analysis set.

Core laboratory interobserver variations were very limited with “almost perfect” correlation coefficients >0.8 for all TTE parameters (ICC for TAPSE 0.900, PASP 0.938, and RV/LV diameter ratio 0.851; see Table 2 ). Interobserver variation did not differ in subgroup analysis between intermediate-risk and low-risk patients (data not shown). Agreement was substantial between core laboratory observers and site-reported data for key parameters (ICC for TAPSE 0.728, PASP 0.726, and RV/LV diameter ratio 0.739; see Table 2 ).

**Table 2:** Agreement between observers and Registro Informatizado Enfermedad TromboEmbolica

	Observer 1 versus 2	*n*	Observers versus RIETE	*n*
TAPSE	0.900 (0.847–0.953)	49	0.728 (0.594–0.862)	47
PASP	0.938 (0.904–0.971)	49	0.726 (0.601–0.852)	45
TRG	0.939 (0.908–0.970)	49	0.919 (0.815–1.000)	10
TAPSE/PASP	0.852 (0.808–0.906)	49	0.851 (0.768–0.934)	43
RV/LV ratio	0.851 (0.785–0.916)	49	0.739 (0.443–1.000)	10
RV diameter	0.876 (0.813, 0.940)	49	N.A.	–
LV diameter	0.79 (0.69, 0.90)	49	N.A.	–

Abbreviations: LV, left ventricular; PASP, pulmonary arterial systolic pressure; RV, right ventricular; RIETE, Registro Informatizado Enfermedad TromboEmbolica; TAPSE, tricuspid annular plane systolic excursion; TRG, tricuspid regurgitation gradient.

Intraclass correlation coefficients between the two independent observers and between an average of the two observers and the RIETE registry. Data are presented as mean (95% confidence interval).

**Fig. 2:**
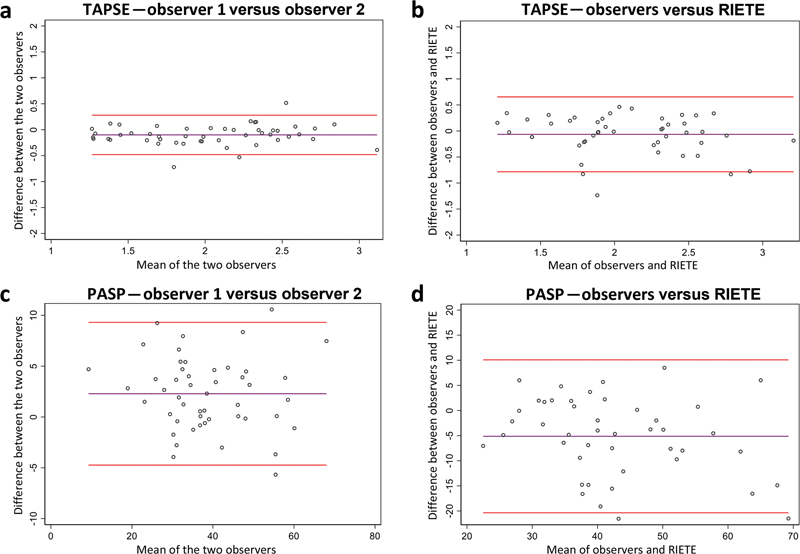
Variation in TAPSE and PASP measurements. Bland–Altman plots for the measurement of TAPSE showing the agreement between the two observers ( **a** ) and between the observers and the RIETE registry ( **b** ). Similarly, for the measurement of PASP with agreement between the two observers ( **c** ) and between observers and RIETE ( **d** ). For interpretation of Bland–Altman plots, bias (purple lines) should be close to zero and 95% Limits of Agreement (red lines) should be as narrow as possible. Furthermore, data points should be evenly distributed around the bias line without any trumpet-shape formation, that is, no systematic difference between the two comparators. For the plots shown here, we note that biases are close to zero, and limits of agreement are smaller between the two core laboratory observers. PASP, pulmonary arterial systolic pressure; RIETE, Registro Informatizado Enfermedad TromboEmbolica; TAPSE, tricuspid annular plane systolic excursion.

**Fig. 3:**
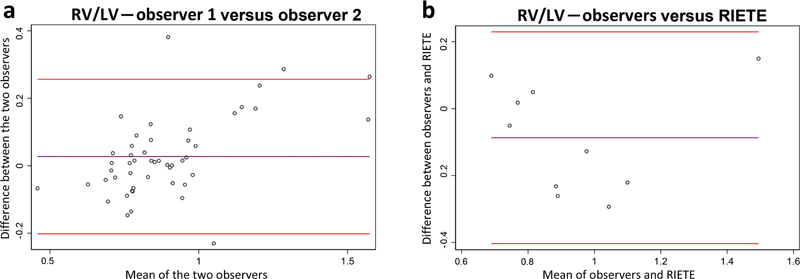
Variation in RV/LV measurements. Bland–Altman plots for the measurement of RV/LV ratio, the agreement between the two observers are shown in ( **a** ) and between observers and RIETE shown in ( **b** ). Bland–Altman interpretation is briefly explained in Fig. 2. For these plots, we note that biases are generally close to zero, but missing data are frequent in the RIETE regTaistry. LV, left ventricular; RIETE, Registro Informatizado Enfermedad TromboEmbolica; RV, right ventricular.

Bland–Altman plots for evaluation of RV function and pulmonary pressure (TAPSE and PASP) are presented in Fig. 2 and plots for RV/LV diameter ratios are presented in Fig. 3 . For the TAPSE/PASP ratio, the bias was −0.01 (95% Limits of Agreement −0.06 to 0.03) between the two observers and 0.00 (95% Limits of Agreement −0.02 to 0.03) for core laboratory analysis versus RIETE.

The categorical agreement of normal versus abnormal RV function between observers and RIETE is shown in Table 3 . Core laboratory echocardiogram interpretations did not result in reclassification of risk category (data not shown).

**Table 3:** Categorical agreement on identification of right ventricular dysfunction

	Observer 1 versus 2			Observer 1 versus RIETE			Observer 2 versus RIETE		
	κ	Percentage	*n*	κ	Percentage	*n*	κ	Percentage	*n*
TAPSE < 17	0.66	85.7	49	0.78	90.6	32	0.76	90.6	32
RV/LV > 1	0.68	89.8	49	0.60	80	10	0.20	60	10
PASP > 36	0.88	93.9	49	0.44	75.6	41	0.47	75.6	41
TRG > 2.9	0.96	93.9	49	0.80	90	10	0.80	90	10
RV diameter ≥ 41 mm	0.62	85.7	49	N.A.			N.A.		

Abbreviations: LV, left ventricular; PASP, pulmonary arterial systolic pressure; RV, right ventricular; RIETE, Registro Informatizado Enfermedad TromboEmbolica; TAPSE, tricuspid annular plane systolic excursion; TRG, tricuspid regurgitation gradient; N.A., not applicable.

Percentage agreement and kappa statistics between the two independent observers and between the two observers and the RIETE registry in the identification of right ventricular dysfunction based on guideline cutoff values. Core laboratory analyses did not cause a change in European Society of Cardiology risk stratification.

## Discussion

**Fig. 4:**
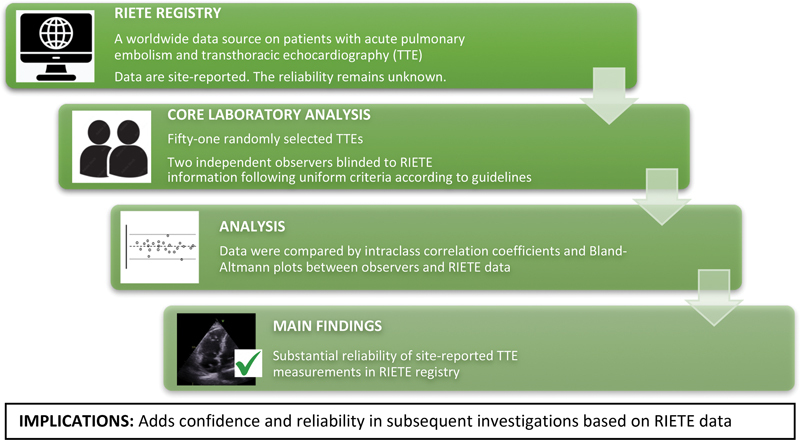
Validation of the RIETE registry by external, blinded core laboratory analysis. The RIETE registry is the world's largest registry on venous thromboembolism including acute pulmonary embolism. The reliability of site-reported echocardiographic measurements was investigated by two blinded, independent observers. This core laboratory analysis confirmed the reliability of site-reported RIETE data and adds confidence in past and future investigations. RIETE, Registro Informatizado Enfermedad TromboEmbolica.

In this study, we validated key echocardiographic measurements of RV function in patients with acute PE from the RIETE registry. The study provides additional information, as such validation study has not been done before and reliability is crucial for the trustworthiness to any register used for research purposes. Agreement was substantial between core laboratory observers and site-reported data for key variables. Our findings support the reliability of echocardiographic data entered in the RIETE registry, especially for TAPSE and PASP ( Fig. 4 ).

### Reliability of Echocardiography in Acute Pulmonary Embolism

Echocardiographic RV dysfunction is associated with increased mortality in PE patients. 14 15 16 Typical TTE findings include RV dilatation, reduced RV systolic function, and increased pulmonary pressure estimates. 1 5 Accordingly, we assessed the reliability of RV/LV diameter ratio, TAPSE and PASP measurements.

Only a few previous PE studies have assessed reliability of TTE readings showing substantial levels of agreement comparable to our reliability assessment of RIETE. 17 18 19 20 Previous investigations were not able to assess the interobserver reproducibility of PASP. 18 Our validation of PASP is a strength of this study despite a larger variation between core laboratory analysis and the RIETE registry ( Fig. 2c, d ). This may be explained by a lack of distinction between PASP and TRPG to many clinicians, though we can only speculate if this was true for the RIETE values. The core laboratory analysis carefully followed the TTE guidelines 8 9 adding between 3 and 15 mm Hg to the TRPG, possibly contributing to the larger variation.

### Reliability of Echocardiography of the Right Ventricle

Evaluation of RV on TTE in general is challenging because of the complex anatomy and function of the ventricle. 3 8 Schnittke et al showed poor interobserver agreement on RV function (kappa −0.05). 21 Taylor and Moore 22 reported higher kappa values among trained physicians compared with residents in the identification of RV strain suggesting that expertise is required to perform the evaluation.

Across several non-PE studies on the reliability of RV echocardiographic parameters, TAPSE measurements often have very high levels of agreement while RV diameter and especially fractional area change (FAC) measurements appear to be less reliable. 23 24 25 26 27 The present study shows the same trend. TAPSE is a simple measurement that clinicians perform regularly, whereas FAC requires more measurements with a higher the risk of variation and compromised reliability.

### Clinical Relevance

The importance of our validation of key RV echocardiographic measurements in the RIETE registry is two-fold: first, since the registry is the world's largest of its kind, it offers important data on acute PE. Several prior studies including TTE information from the registry have already been published. 28 29 30 31 The current analysis provides support for the findings of these previous studies by validating site-reported echocardiogram findings against a blinded core laboratory analysis. It also provides reassurance that site-reported TTE can be relied upon for European Society of Cardiology risk classification. Second, the interrater correlation of echocardiographic measures has clinical relevance, since RV dysfunction contributes to clinical decision-making, including treatment escalation (e.g., to surgery or thrombolysis). Confirming that, despite some variation, interpretation of echocardiographic parameters by different readers leads to fairly consistent classifications of risk has important clinical implications.

## Limitations

Some limitations should be considered. Data from two RIETE sites were included in this core laboratory validation analysis out of 133 active hospitals enrolling patients. These two sites encompass a large proportion of patients in RIETE (8.6%). We acknowledge that it may affect generalizability, but was a pragmatic choice because of the difficulty in obtaining individual agreements for transfer of echocardiograms. Inclusion of large volume centers only may, however, affect generalizability to other RIETE centers with less ability to maintain echocardiographic competences. Nevertheless, echocardiographic evaluation of RV function in PE relies on standard measurements, like those included in the present study, which can be easily obtained by all operators with basic echocardiographic training. All sites had local operators, so we do not suspect including other sites would deteriorate the validity of the findings. Second, among the thousands of patients with PE enrolled in RIETE, relatively few were used in this validation study. However, the study sample size was based on an a priori power calculation. In fact, prior analyses of TTE validation used smaller samples for this purpose. 17 23 26 Third, despite some variability between the observers, we did compare an average of the two observers' measurements to the RIETE registry data. This appears reasonable with a bias close to zero and ICC > 0.9. As this study is based on reassessment from already collected images, time is not an issue, and it is important to emphasize that the RIETE registry is a prospective registry. We acknowledge that variation may exist between echocardiographers in terms of both image acquisition and interpretation, and this study did not involve de novo image acquisition through a central laboratory but rather, involved a core laboratory to review and interpret the images that were already acquired using standardized and predefined criteria by the core laboratory. De novo image acquisition from each patient twice is extremely resource intensive and to our knowledge, has not been performed in any prior multicenter PE study. Our study is limited to the validation of the interpretation of previously acquired echocardiogram images. Finally, data on RV diameter were frequently missing, forcing us to remove that variable, and the study is underpowered for detection of changes in RV/LV ratio. However, there were sufficient data to show reliability of especially TAPSE and PASP values.

## Conclusion

We showed substantial reliability of site-reported key RV echocardiographic measurements in the RIETE registry. Reliability was especially robust for TAPSE and PASP. Ascertaining the validity of such data adds confidence and reliability for subsequent investigations.
