# Supplementary material for: Validation of Echocardiographic Measurements in Patients with Pulmonary Embolism in the RIETE Registry
Source: TH Open. 2024 Jan 8;8(1):e1–8. doi: 10.1055/s-0043-1777765 (PMC10774011; doi:10.1055/s-0043-1777765)
Supplement: Supplementary file 1 — Supplementary Material [file 10-1055-s-0043-1777765-s23090040.pdf]

Supplementary Materials

Coordinator of the RIETE Registry: Manuel Monreal

RIETE Steering Committee Members: Paolo Prandoni, Benjamin Brenner, and Dominique Farge-Bancel

RIETE National Coordinators: Raquel Barba (Spain), Pierpaolo Di Micco (Italy), Laurent Bertoletti (France), Sebastian Schellong (Germany), Inna Tzoran (Israel), Abilio Reis (Portugal), Marijan Bosevski (Republic of Macedonia), Henri Bounameaux (Switzerland), Radovan Malý (Czech Republic), Peter Verhamme (Belgium), Joseph A. Caprini (United States), Hanh My Bui (Vietnam)

RIETE Registry Coordinating Center: S&H Medical Science Service

Members of the RIETE Group

SPAIN: **Adarraga MD, Alberich-Conesa A, Aibar J, Alda-Lozano A, Alonso-Carrillo J, Alfonso J, Amado C, Angelina-García M, Arcelus JI, Ballaz A, Barba R, Barbagelata C, Barreiro B, Barrón M, Barrón-Andrés B, Beddar-Chaib F, Blanco-Molina A, Botella E, Cañas I, Carrero R, Castellanos G, Chasco L, Claver G, Criado J, del Toro J, De Ancos C, Demelo-Rodríguez P, De Juana-Izquierdo C, Díaz-Pedroche MC, Díaz-Peromingo JA, Dubois-Silva A, Escribano JC, Falgá C, Farfán-Sedano AI, Fernández-Aracil C, Fernández-Capitán C, Fernández-Jiménez B, Fernández-Reyes JL, Fidalgo MA, Font C, Francisco I, Gabara C, Galeano-Valle F, García-González C, García-Bragado F, García-Ortega A, Gavín-Sebastián O, Gil-De Gómez M, Gil-Díaz A, Gómez-Cuervo C, González-Martínez J, Grau E, Guirado L, Gutiérrez J, Hernández-Blasco L, Jara-Palomares L, Jiménez D, Jou I, Joya MD, Lecumberri R, León-Ramírez JM, Llamas P, Lobo JL, López-De la Fuente M, López-Jiménez L, López-Miguel P, López-Núñez JJ, López-Ruiz A, López-Sáez JB, Lorente MA Lorenzo A, Lumbierres M, Madridano O, Maestre A, Marchena PJ, Marcos M, Martín del Pozo M, Martín-Martos F, Maza JM, Mercado MI, Monreal M, Muñoz-Gamito G, Navas MS, Nieto JA, Nomdedeu M, Núñez-Fernández MJ, Olid M, Ortiz M, Otálora S, Otero R, Pacheco-Gómez N, Pagan J, Paredes-Mariñas E, Parra P, Parra-Rosado P, Pedrajas JM, Pérez-Ductor C, Pérez-Pinar M, Peris ML, Pesce ML, Porras JA, Poyo-Molina J, Puchades R, Riera-Mestre A, Rivera-Civico F, Rivera-Gallego A, Rodríguez-Cobo A, Rosa V, Romero-Brugera M, Rubio CM, Ruiz-Artacho P, Ruiz-Giménez N, Ruiz-Ruiz J, Salgueiro G, Sancho T, Sendín V, Sigüenza P, Soler S, Suárez-Fernández S, Tirado R, Tolosa C, Torrents-Vilar A, Torres MI, Trujillo-Santos J, Uresandi F, Valle R, Vidal G, Villalobos A, Villares P, Zamora C**

AUSTRIA: **Ay C, Nopp S, Pabinger I**
BELGIUM: **Vanassche T, Verhamme P, Verstraete A**
BRAZIL: **Yoo HHB**
COLOMBIA: **Argüello JD, Montenegro AC, Roa J**
CZECH REPUBLIC**: Hirmerova J, Malý R**
FRANCE: **Accassat S, Bertoletti L, Bura-Riviere A, Catella J, Chopard R, Couturaud F, Espitia O, Grange C, Leclercq B, Le Mao R, Mahé I, Moustafa F, Plaisance L, Poenou G, Sarlon-Bartoli G, Suchon P, Versini E**
GERMANY: **Schellong S**
ISRAEL: **Brenner B, Dally N, Tzoran I**
IRAN: **Sadeghipour P**
ITALY: **Abenante A, Barillari G**, **Basaglia M, Bilora F, Bortoluzzi C, Brandolin B, Ciammaichella M, Colaizzo D, Dentali F, Di Micco P, Grandone E, Imbalzano E, Marcon C, Negro F, Pesavento R, Poz A, Prandoni P, Scarinzi P, Siniscalchi C, Taflaj B, Tufano A, Visonà A, Vo Hong N, Zalunardo B**
LATVIA: **Kigitovica D, Skride A, Strautmane S**
PORTUGAL: **Fonseca S, Manuel M, Meireles J, Pinto S**
REPUBLIC OF MACEDONIA: **Bosevski M**
SWITZERLAND**: Bounameaux H, Mazzolai L**
UNITED KINGDOM: **Aujayeb A**
UNITED STATES: **Caprini JA, Weinberg I**
VIETNAM**: Bui HM, Nguyen A**

Acknowledgments

We express our gratitude to **SANOFI** and **ROVI** for supporting this Registry with an unrestricted educational grant. We also thank the RIETE Registry Coordinating Center, S&H Medical Science Service, for their quality control data, logistics, and administrative support.

 © 2024. The Author(s).
